# Positive end-expiratory pressure improves elastic working pressure in anesthetized children

**DOI:** 10.1186/s12871-018-0611-8

**Published:** 2018-10-24

**Authors:** Pablo Cruces, Sebastián González-Dambrauskas, Federico Cristiani, Javier Martínez, Ronnie Henderson, Benjamin Erranz, Franco Díaz

**Affiliations:** 1 Pediatric Intensive Care Unit, Hospital El Carmen de Maipú, Santiago, Chile; 2 Centro de Investigación de Medicina Veterinaria, Escuela de Medicina Veterinaria, Facultad de Ecología y Recursos Naturales, Universidad Andres Bello, Santiago, Chile; 3 Pediatric Intensive Care Unit, Centro Hospitalario Pereira Rossell, Montevideo, Uruguay; 4 Department of Anesthesiology, Centro Hospitalario Pereira Rossell, Montevideo, Uruguay; 5 Unidad de Cuidados Intensivos Pediátricos, Clínica Alemana de Santiago, Avda. Vitacura, 5951 Santiago, Chile; 6 Facultad de Medicina Clínica Alemana Universidad del Desarrollo, Santiago, Chile; 7 Area de Cuidados Críticos, Hospital Padre Hurtado, Santiago, Chile

**Keywords:** Positive end-expiratory pressure, Mechanical ventilation, Respiratory mechanics, Pediatrics

## Abstract

**Background:**

Positive end-expiratory pressure (PEEP) has been demonstrated to decrease ventilator-induced lung injury in patients under mechanical ventilation (MV) for acute respiratory failure. Recently, some studies have proposed some beneficial effects of PEEP in ventilated patients without lung injury. The influence of PEEP on respiratory mechanics in children is not well known. Our aim was to determine the effects on respiratory mechanics of setting PEEP at 5 cmH_2_O in anesthetized healthy children.

**Methods:**

Patients younger than 15 years old without history of lung injury scheduled for elective surgery gave informed consent and were enrolled in the study. After usual care for general anesthesia, patients were placed on volume controlled MV. Two sets of respiratory mechanics studies were performed using inspiratory and expiratory breath hold, with PEEP 0 and 5 cmH_2_O. The maximum inspiratory and expiratory flow (Q_I_ and Q_E_) as well as peak inspiratory pressure (PIP), plateau pressure (P_PL_) and total PEEP (tPEEP) were measured. Respiratory system compliance (C_RS_), inspiratory and expiratory resistances (RawI and RawE) and time constants (K_TI_ and K_TE_) were calculated. Data were expressed as median and interquartile range (IQR). Wilcoxon sign test and Spearman’s analysis were used. Significance was set at *P* < 0.05.

**Results:**

We included 30 patients, median age 39 (15–61.3) months old, 60% male. When PEEP increased, PIP increased from 12 (11,14) to 15.5 (14,18), and C_RS_ increased from 0.9 (0.9,1.2) to 1.2 (0.9,1.4) mL·kg^− 1^·cmH_2_O^− 1^; additionally, when PEEP increased, driving pressure decreased from 6.8 (5.9,8.1) to 5.8 (4.7,7.1) cmH_2_O, and Q_E_ decreased from 13.8 (11.8,18.7) to 11.7 (9.1,13.5) L·min^− 1^ (all *P* < 0.01). There were no significant changes in resistance and Q_I_.

**Conclusions:**

Analysis of respiratory mechanics in anesthetized healthy children shows that PEEP at 5 cmH_2_O places the respiratory system in a better position in the P/V curve. A better understanding of lung mechanics may lead to changes in the traditional ventilatory approach, limiting injury associated with MV.

**Electronic supplementary material:**

The online version of this article (10.1186/s12871-018-0611-8) contains supplementary material, which is available to authorized users.

## Background

There are many detrimental effects of positive pressure mechanical ventilation (MV) to the lung parenchyma, giving shape to the entity we know as ventilator induced lung injury (VILI) [1, 2]. Despite that it was initially described for injured lungs [3], VILI has been recognized to affect patients with uninjured lungs, triggering many pathways of local and systemic inflammation [1–4]. Positive pressure MV can cause VILI even when applied for short periods of time, and the role of protective MV during anesthesia has become important for preventing postoperative complications [5–10]. Although the exact incidence of VILI during general anesthesia is unknown, the lungs of patients under general anesthesia are especially vulnerable to VILI, since anesthetic induction reduces the end expiratory lung volume (EELV) by 9–25% in adults and up to 46% in children [11–15]. Cyclic opening and collapsing of alveoli have been indicated as one of the primary mechanisms of VILI during anesthesia. Imaging studies have shown that general anesthesia induces atelectasis of dependent regions and that the use of PEEP prevents its formation [3, 5, 9, 10, 14]. Protective ventilation during general anesthesia [16, 17] to limit tidal volume (6–8 ml/kg) has been widely accepted and incorporated into the operating room (OR), but PEEP use is still not a common practice for patients undergoing general anesthesia [6–10, 17, 18].

In the absence of respiratory muscle activity, working pressure of the respiratory system is the pressure needed to overcome frictional forces, elastic forces and impedance. In this way, an improvement in CRS reflects lower elastic work pressures and, therefore, a pending situation of its more favorable dynamic pressure/volume curve (P/V curve) [19]. These observations also have been described in pediatric animal models [20, 21].

There is little evidence to guide MV during general anesthesia in children [18, 22, 23]. MV parameters are primarily dependent on anesthesiologist preferences and on the characteristics of anesthesia machines [24]. Facing this scenario, many scientific societies have pointed as priority areas of research to the pathophysiology, respiratory mechanics and practice of MV in children for improving outcomes and reducing secondary long-term lung injury [23].

With these facts in mind, we sought to compare respiratory mechanics of setting PEEP at 5 cmH_2_O compared to ZEEP (PEEP set at 0 cmH_2_O) in anesthetized children without acute pulmonary pathology undergoing elective surgery. We hypothesized that usual respiratory mechanics parameters, measured with a commercial mechanical ventilator, would identify a more protective ventilation with addition of PEEP 5 cmH_2_O. Special emphasis was placed on evaluation on respiratory work pressure, flow patterns, resistance and inspiratory and expiratory time constants.

## Methods

### Study design and setting

This prospective study was conducted at the Surgical Block of Centro Hospitalario Pereira Rossell located in Montevideo, Uruguay. The local ethic committee approved the study and informed consent was obtained for each patient before entering the OR.

### Study population

Between February 1, 2015 and April 30, 2016, children younger than 15 years old without preexisting lung injury scheduled for elective surgery were screened for the study. For definitive selection, patients categorized as ASA I or II, requiring orotracheal intubation for the surgery according to the anesthesiologist’s criteria, were considered. Patients were excluded if they had any acute condition before or during anesthesia (e.g., laryngospasm, bronchoconstriction, pneumothorax), thoracic surgery, thoracic or airway malformation and chronic lung disease with oxygen dependence. Additionally, patients with endotracheal tube air leak > 20% of tidal volume (V_T_) were excluded due to possible interference with data acquisition.

### Data collection

We registered demographics and clinical information at admission. All procedures before respiratory system mechanics measurements were done according to institutional protocol and anesthesiologist preferences, including anesthesia induction, orotracheal intubation and initial ventilatory settings on the anesthesia machine.

### Study protocol

Patients were ventilated on a Galileo Gold® ventilator (Hamilton, Bonaduz, Switzerland) on volume control mode after verification of correct positioning of the endotracheal tube. Baseline settings were as follows: V_T_ = 6–8 mL·kg^− 1^, PEEP = 5 cmH_2_O, fraction of inspired oxygen (FiO_2_) was adjusted to a target pulse oximetry greater than 95%, inspiratory: expiratory ratio = 1:2, and respiratory rate (RR) was adjusted to achieve an end-tidal carbon dioxide (ETCO_2_) 40 ± 5 mmHg. Tracheal tube leak compensation was deactivated through the measurements.

### Respiratory mechanics measurements

Two sets of measurements were performed, at ZEEP and PEEP 5 cmH_2_O, separated by 5 min of stability, following local protocol for respiratory mechanics measurements. This protocol is summarized in Additional file 1 [19]. All measurements were made in pre-incision surgical time.

Ventilator parameters [peak inspiratory pressure (PIP), plateau pressure (P_PL_), extrinsic (set) PEEP (PEEP), total PEEP (tPEEP), intrinsic PEEP (iPEEP = tPEEP - PEEP), driving pressure (∆P = P_PL_–tPEEP), mean airway pressure expiratory (Paw), V_T_, inspiratory time (IT), respiratory rate (RR), maximum inspiratory and expiratory flow (Q_I_ and Q_E_, L·min^− 1^)] were assessed. An inspiratory hold followed by an expiratory hold was performed following the protocol described in Additional file 1. Flow and pressure parameters in these quasi-static conditions at the Y piece (*proximal flow sensor*) were recorded in an ad hoc Microsoft Excel 2010 (Microsoft®, NY, USA) database to calculate respiratory system compliance (C_RS_, mL·cmH_2_O^− 1^·kg^− 1^), inspiratory and expiratory airway resistance (RawI and RawE, cmH_2_O·L^− 1^·s^− 1^), and inspiratory and expiratory time constants (K_TI_ and K_TE_, s) according to formulas described in Additional file 2.

### Data analysis

Data are expressed as median and interquartile range (IQR). A previous study found that changing PEEP from 0 to 12 cmH_2_0 resulted in a decrease in dynamic compliance (Cdyn) in 9.4 ml/cmH_2_O with SD 6.8 [25]. Given the fact that our protocol included a moderate modification of PEEP, from 0 to 5 ml/cmH_2_O, we expected a smaller change of Cdyn, 2/3 of previously described [25]. A sample size of 30 patients is needed to determine a variation of Cdyn by 6 ml/cmH_2_O, using chi-square test and assuming an α of 0.05 and a power of 90%. Normality was assessed with the Anderson–Darling test. Wilcoxon sign test was performed for comparisons between respiratory mechanics assessments. Spearman’s analysis was used to examine correlations between changes in respiratory mechanics and age and ideal body weight. Differences were considered significant if *p* < 0.05. All statistical analyses were performed using SPSS 20.0 (SPSS Inc., Chicago, IL, USA). Figures were plotted with GraphPad Prism version 5.0c for Mac (GraphPad Software, La Jolla, CA, USA).

## Results

Thirty patients were included in the study. Sixty percent were male, the median age was 39 months (15–61.3), and weight was 15 kg (10.6–21).

Sixty percent of patients received inhaled anesthesia and 40% mixed inhalation and intravenous anesthesia. Abdominal surgery was the most frequent (20 cases), mostly hernioplasty. Malformation of the digestive tract was the most frequent comorbidity, present in 7 patients. Table 1 shows clinical characteristics of included patients.

**Table 1:** Clinical characteristics of children included in the study

Number	Age (mo)	Weight (kg)	Height (cm)	Surgery	Comorbidities
1	40–50	17.5	105	Inguinal hernia	none
2	40–50	16.7	105	Umbilical hernia	none
3	30–40	13	90	Cryptorchid	none
4	10–20	5.1	67	CVL placement	Short bowel insufficiency
5	30–40	15	94	Anorectoplasty	Partial anomalous venous return
6	40–50	15	94	Inguinal hernia	Asthma
7	20–30	11.2	82	Epigastric hernia	none
8	10–20	9.2	70	Enterostomy closure	Imperforated anus
9	50–60	18	101	Enterostomy closure	Colostomy
10	60–70	14.2	104	Esophageal dilatation	Esofageal coloplasty
11	50–60	26	114	Abdominal tumor	none
12	30–40	22	99	Hidrocele	none
13	50–60	19	90	Fimosis	none
14	30–40	23	103	Cryptorchid	none
15	20–30	11.9	81	Inguinal hernia	none
16	70–80	24.6	120	Cryptorchid	none
17	3–10	7	63	Inguinal hernia	none
18	10–20	10.4	77	Inguinal hernia	none
19	10–20	13.5	80	Cryptorchid	none
20	120–130	25.8	132	Colecystectomy	none
21	3–10	5.7	62	Inguinal hernia	none
22	140–150	40	154.5	Thyroidectomy	none
23	3–10	4.9	N/A	Eventration repair	Traumatic eventration
24	80–90	19.7	116	Enterostomy closure	Hirschprung’s Disease
25	80–90	27	118	Enterostomy closure	Hirschprung `sDisease
26	110–120	29	129.5	Anoplasty	Anorectal Malformation
27	100–110	21	116	CVL placement	Astrocytoma
28	10–50	14	103	Anoplasty	Anorectal malformation
29	1–10	4.6	56.5	Exploratory laparoscopy	none
30	10–20	7.3	77	CVL placement	Lymphoblastic leukemia

*mo* months old, *CVL* central venous line, *N/A* non-available

**Fig. 1:**
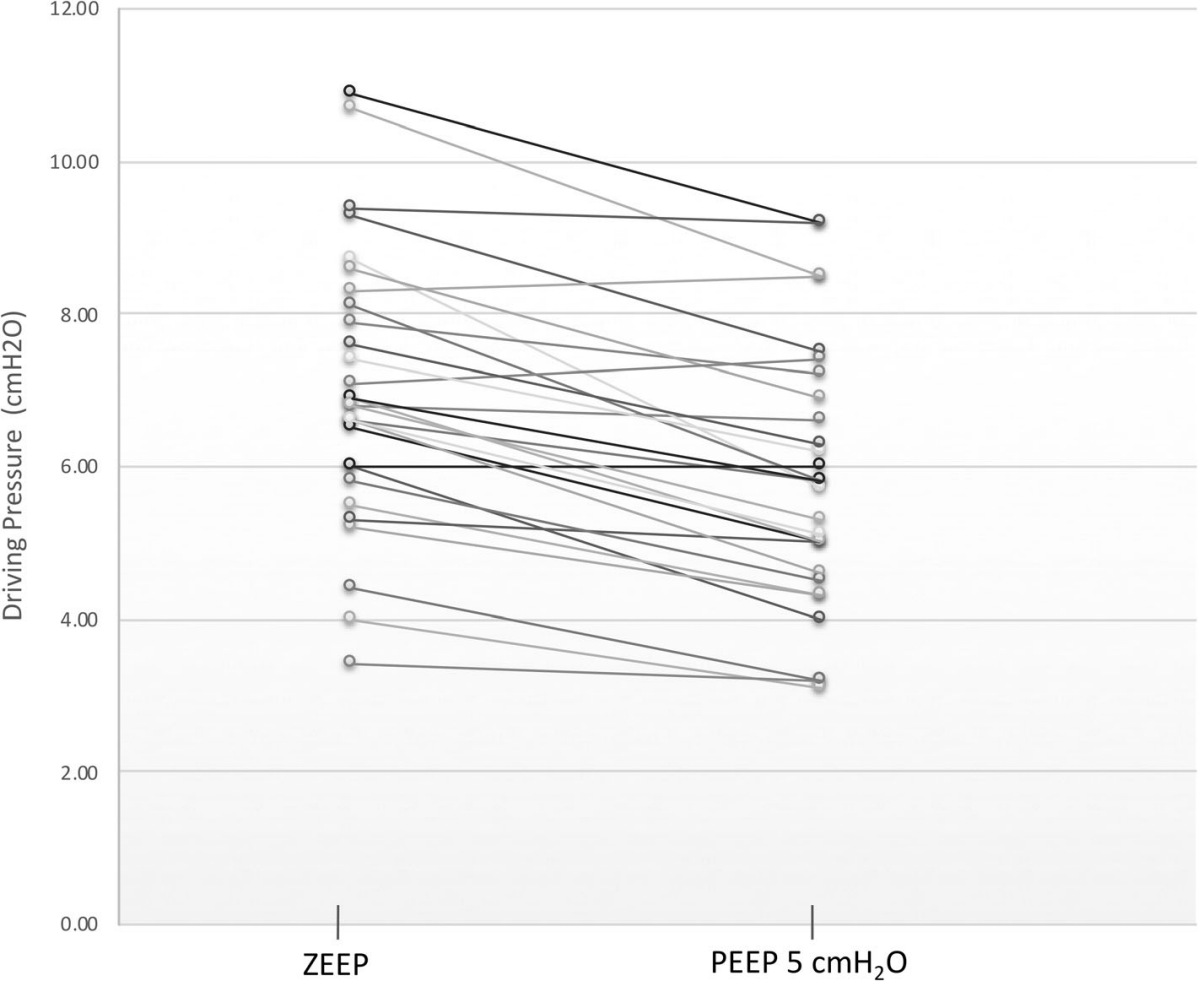
Individual value plot of driving pressure (cmH_2_O) with ZEEP and PEEP at 5 cmH_2_O in anesthetized healthy children

There were no complications related to the protocol. Table 2 shows ventilatory parameters and respiratory system mechanics for the study population with ZEEP and PEEP of 5 cmH_2_O. After setting PEEP at 5 cmH_2_O we observed an increase in PIP, P_PL_ and Paw, with a concomitant decrease in iPEEP and ∆P. Figure 1 shows individual changes on ∆P throughout the study. No modifications were done on V_T_, and thus, changes on ∆P were coupled to variation on C_RS_.

**Table 2:** Ventilatory parameters and respiratory system mechanics of children under general anesthesia with ZEEP and PEEP of 5 cmH_2_O

	ZEEP (*n* = 30)		PEEP 5 (*n* = 30)		*P* value
	MEDIAN	P25, P75	MEDIAN	P25, P75	
FiO_2_	0.40	0.36, 0.50	0.40	0.36, 0.50	1.000
V_TE_	6.79	6.18, 7.36	6.53	5.97, 7.20	0.290
RR	24	21, 26	24	21, 26	1.0
IT	0.85	0.72, 1.02	0.85	0.72, 1.03	1.00
PIP	12	11, 14	15.5	14.0, 18.5	< 0.01
P_PL_	7.9	7.2, 9.18	10.95	9.7, 12.6	< 0.01
Paw	4.1	3.6, 4.9	8.5	7.9, 9.8	< 0.01
Q_I_	11	8.1, 13.1	11	8.1, 13.1	0.317
Q_E_	13.8	11.8, 13.1	11.7	9.1, 13.5	< 0.01
RawI	25.7	18.6, 34.3	26.4	20.1, 33.1	0.447
RawE	28.9	21.9, 39.4	29.3	22.3, 42.1	0.629
C_RS_	0.96	0.89, 1.22	1.19	0.94, 1.39	< 0.01
K_TI_	0.36	0.31, 0.48	0.45	0.38, 0.59	0.004

*ZEEP* PEEP 0 cmH_2_O, *FiO*_*2*_ fraction of inspired oxygen, *V*_*TE*_ expiratory tidal volume (mL·kg^−1^), *RR* respiratory rate (breath per minute), *IT* inspiratory time (s), *PIP* peak inspiratory pressure (cmH_2_O), *P*_*PL*_ plateau pressure (cmH_2_O), *Paw* mean airway pressure (cmH_2_O), *Q*_*I*_ peak inspiratory flow (L·min^− 1^), *Q*_*E*_ peak expiratory flow, (L·min^− 1^), *RawI* inspiratory airway resistance (cmH_2_O·L^− 1^·s^− 1^), *RawE* expiratory airway resistance (cmH_2_O·L^− 1^·s^− 1^), *C*_*RS*_ static respiratory system compliance (mL·cmH_2_O^− 1^·kg^− 1^), *K*_*TI*_ inspiratory time constant (s)

There was a moderate correlation between age and ideal body weight and changes in Q_E_ and changes in K_TE_. Additional file 3 shows the correlation between changes in respiratory parameters and age and ideal body weight. No other correlations with age and weight were found.

## Discussion

**Fig. 2:**
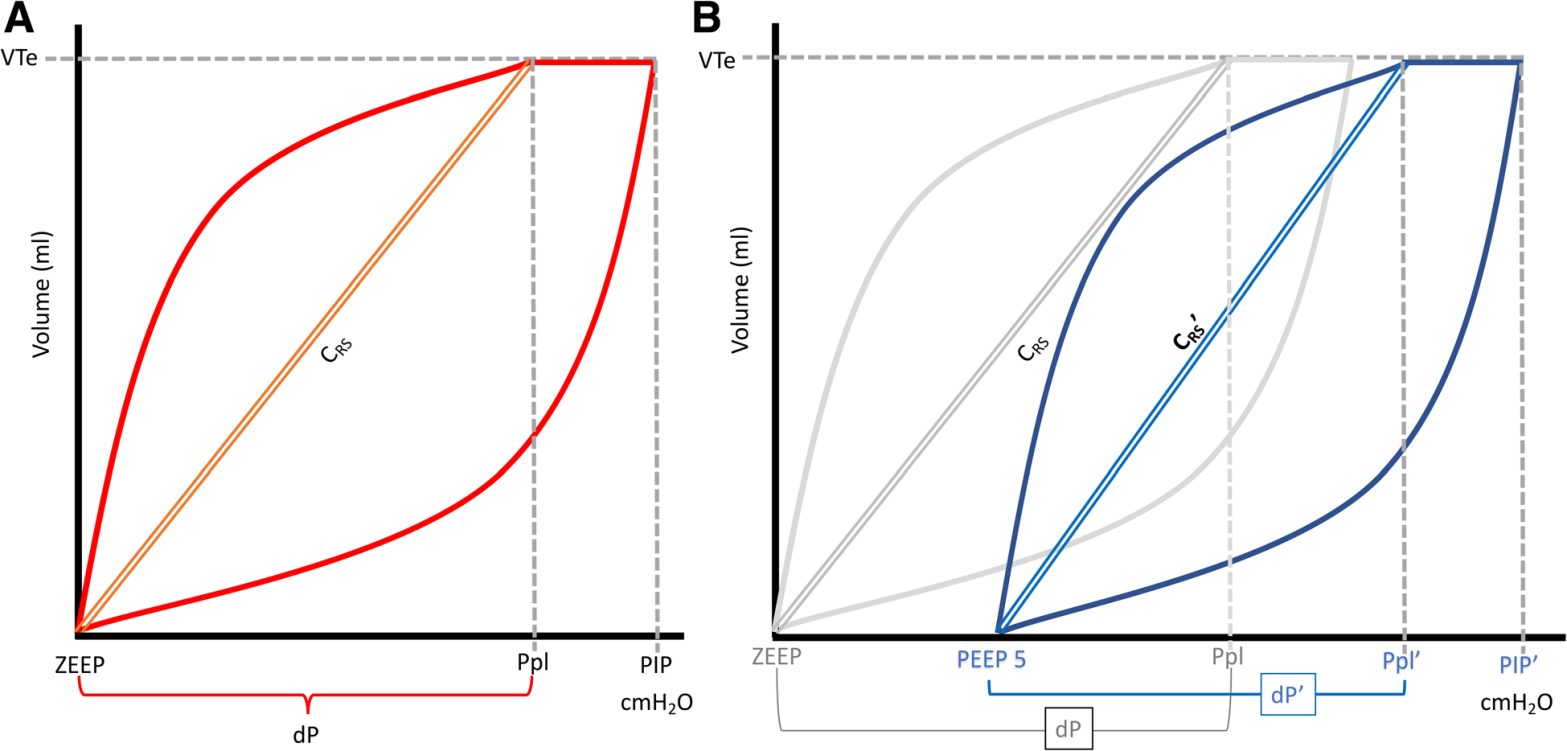
Pressure – Volume loop (P/V curve) sketch summarizing findings of the study: Anesthetized children with ZEEP (panel **a**) and after setting PEEP at 5 cmH_2_O (panel **b**). PIP, P_PL_ and Paw increased after addition of PEEP of 5 cmH_2_O (PIP’, P_PL_’,Paw’), but dP decreased (dP'). Given the same V_T_, C_RS_, represented as the angle of the diagonal line between PEEP and P_PL_, increased (C_RS_’). All of them are sign of a more efficient working pressures of the respiratory system better position in P/V curve. On (panel **b**) respiratory mechanics with ZEEP was added in gray tone as reference

In this study, we measured pulmonary mechanics in anesthetized healthy children on MV for elective surgery at ZEEP and PEEP 5 cmH_2_O using a commercially available mechanical ventilator. Setting PEEP at 5 cmH_2_O resulted in a significant decrease in ∆P and iPEEP, thereby improving C_RS_. Changes observed in respiratory mechanics after setting PEEP at 5 cmH_2_O are summarized in Fig. 2.

We also found a decrease in Q_E_ when PEEP was applied. All these findings are indirect signs of lungs in a better position in dynamic P/V curve, suggesting that the addition of a low level of PEEP improves respiratory mechanics.

The effect of PEEP on patients with ARDS was described more than a decade ago, promoting recruitment of non-aerated lung volume and increasing EELV. More recently, many investigators have shown that PEEP should be incorporated to lung protective strategy during the perioperative period in patients at risk for ARDS as well as previously healthy patients [6, 8–10]. In this setting, hypoxemia was not associated with the reduction in EELV, and thus, hypoxemia is thought to be a poor predictor of potential injurious MV. Similar findings have been described in children. Serafini et al. described densities in dependent regions of both lungs on CT scan of 10 infants after induction of anesthesia. They observed reopening of the collapsed lung with addition of 5 cmH_2_O of PEEP for 5 min [14]. Kadini et al., in a small study of 8 anesthetized children (range between 2.5 and 6.5 years old), described that 5 cmH_2_O resulted in a significant increase of VT; thus, C_RS_ improved [24].

Our results show that the addition of PEEP sets the respiratory system in a better position of the P/V curve, probably related to the reduction of the atelectasis in lung dependent zones after induction of anesthesia and MV with ZEEP. An increase in EELV, maintaining the same V_T,_ denotes a reduction in global strain and, potentially, VILI [26].

∆P has taken high priority after the recent pooled data analysis of adult patients with ARDS that showed a significant relationship with mortality [27]. ∆P represents the pressure required for the movement of inspiratory flow and depends on the lung and chest wall viscoelastic resistance [28]. Recently, Neto et al. in a meta-analysis of 2250 patients under general anesthesia found that high ∆P was the only MV parameter associated with postoperative complications [29]. Even more, they found that changes in PEEP that resulted in an increase in ∆P were associated with more postoperative pulmonary complications. It is not surprising that in these studies, ∆P was the best predictor of unfavorable outcomes. We believe that the best performance of this parameter is because it integrates the set tidal volume and the patient’s compliance, thus giving a more precise idea of the individual conditions of each patient.

In our patients, we observed a 14.8% (CI95% 9.3,20.3%) reduction of ∆P when PEEP of 5 cmH_2_O was applied. The range of improvement was very wide and only one patient had a significant increase in ∆P (greater than 10%). These results are in accordance with a study by Wirth et al. They elegantly showed, with electric impedance tomography in 30 anesthetized children, that changing PEEP from 2 cmH_2_O to 5 cmH_2_O homogenizes regional lung ventilation [30]. One of our patients had a significant increase of ∆P. This a very good example that ventilatory settings need to be tailored individually, because adding 5 cmH_2_0 of PEEP probably led to overdistension in this patient. We maintained the set V_T_ after applying PEEP of 5 cmH_2_O; thus, the reduction of ∆P was dependent on the improvement in C_RS_. The improvement in C_RS_ and EELV may be seen as minor, but we believe that even these small changes may have significant consequences during and after surgery, reducing lung inflammation, alteration of gas exchange, among others. Experimental and clinical data have shown that injurious ventilatory parameters can generate detrimental effects, even when applied for short periods of time [1, 2, 31, 32].

The prolongation of K_TI_ and K_TE_ with PEEP of 5 cmH_2_O probably reflects mathematical coupling of changes on C_RS_ without modifications on airway resistance. In the same way, the observed lower expiratory flow is related to the lower ∆P. These parameters are indirect markers of improvement on respiratory system mechanics and more protective ventilation.

Our study has some limitations. Respiratory mechanics measurements were done before the surgery, after a short period of applied PEEP; thus, we cannot extrapolate these findings to other surgical timings, e.g., during pneumoperitoneum, when the effect of PEEP preventing lung collapse could be even higher. Due to small size of patients, we did not measure pleural pressure, so we could not determine the contribution of the chest wall to C_RS_. Included patients were heterogeneous and age range is wide, so in the absence of normal reference data, we cannot generalize these results to all pediatric patients (i.e., younger patients have higher chest compliance, being at higher risk for derecruitment of the lung on ZEEP). Finally, we have to acknowledge that setting of MV parameters can directly modify the component of the equation of motion (i.e., Q_I_, RR, I:E ratio), but we tried to standardize the ventilatory setting during measurements. Despite these limitations, we consider our observation of the effect of PEEP in anesthetized children under mechanical ventilation important in terms of a pathophysiological approach to reduce VILI.

## Conclusion

Setting PEEP at 5 cmH_2_O in children during general anesthesia improved elastic working pressure of the respiratory system, decreasing driving pressure and intrinsic PEEP. These findings are indirect signs of lungs in a better position in pressure/volume curve, suggesting that the addition of a low level of PEEP improves respiratory mechanics. These findings may be measured with usual mechanical ventilators and anesthesia machines, analyzing respiratory system mechanics after an inspiratory and expiratory hold. Future studies in infants are needed to address respiratory mechanics during anesthesia, ideally in a specific age group and pathologies with high-risk postoperative complications. A better understanding of respiratory system mechanics in children during general anesthesia may lead to a better titration of mechanical ventilation, preventing VILI.

## Additional files

Additional file 1: Respiratory mechanics measurements. Panel A shows Respiratory mechanics measurement protocol. Panel B shows and illustration of Airway Pressure versus time and flow versus time curves during inspiratory and expiratory breathhold. The components of work of breathing, elastic and threshold are represented. (JPG 2451 kb)

Additional file 2: Formulas for estimation of lung mechanics in quasi - static conditions. (DOCX 15 kb)

Additional file 3: Correlations between changes in respiratory parameters and age and ideal body weight. (DOCX 15 kb)

## Abbreviations

ARDS: Acute respiratory distress syndrome
C_RS_: Compliance respiratory system
CT: Computed tomography
EELV: End expiratory lung volume
iPEEP: Intrinsic positive pressure at the end of exhalation
K_TE_: Expiratory time constant
K_TI_: Inspiratory time constant
MV: Mechanical ventilation
Paw: Airway pressure
PEEP: Positive pressure at the end of exhalation
PIP: Peak inspiratory pressure
P_PL_: Plateau pressure
Q_E_: Maximum expiratory flow
Q_I_: Maximum inspiratory flow
RawE: Resistance expiratory airway
RawI: Resistance inspiratory airway
RR: Respiratory rate
tPEEP: Total positive pressure at the end of exhalation
VILI: Ventilator induced lung injury
V_T_: Tidal volume
ZEEP: Positive pressure at the end of exhalation set at 0 cmH_2_O
ΔP: Driving pressure

## References

[1] Slutsky AS, Ranieri VM (2013). Ventilator-induced lung injury. N Engl J Med.

[2] Donoso A, Cruces P (2007). Daño pulmonar inducido por ventilación mecánica y estrategia ventilatoria convencional protectora. Rev Chil Pediatr.

[3] Bein T, Grasso S, Moerer O, Quintel M, Guerin C, Deja M (2016). The standard of care of patients with ARDS: ventilatory settings and rescue therapies for refractory hypoxemia. Intensive Care Med.

[4] Imai Y, Kawano T, Miyasaka K, Takata M, Imai T, Okuyama K (1994). Inflammatory chemical mediators during conventional ventilation and during high frequency oscillatory ventilation. Am J Respir Crit Care Med.

[5] Tusman G, Böhm SH, Warner DO, Sprung J (2012). Atelectasis and perioperative pulmonary complications in high-risk patients. Curr Opin Anaesthesiol.

[6] Vargas M, Sutherasan Y, Gregoretti C, Pelosi P (2014). PEEP role in ICU and operating room: from pathophysiology to clinical practice. ScientificWorldJournal.

[7] Futier E, Constantin JM, Paugam-Burtz C, Pascal J, Eurin M, Neuschwander A (2013). IMPROVE study group. A trial of intraoperative low-tidal-volume ventilation in abdominal surgery. N Engl J Med.

[8] Wolthuis EK, Choi G, Dessing MC, Bresser P, Lutter R, Dzoljic M (2008). Mechanical ventilation with lower tidal volumes and positive end-expiratory pressure prevents pulmonary inflammation in patients without preexisting lung injury. Anesthesiology.

[9] Barbosa FT, Castro AA (2014). de Sousa-Rodrigues CF. positive end-expiratory pressure (PEEP) during anaesthesia for prevention of mortality and postoperative pulmonary complications. Cochrane Database Syst Rev.

[10] Severgnini P, Selmo G, Lanza C, Chiesa A, Frigerio A, Bacuzzi A (2013). Protective mechanical ventilation during general anesthesia for open abdominal surgery improves postoperative pulmonary function. Anesthesiology.

[11] Wahba RW (1991). Perioperative functional residual capacity. Can J Anaesth.

[12] Fletcher ME, Stack C, Ewart M, Davies CJ, Ridley S, Hatch DJ (1985). Respiratory compliance during sedation, anesthesia, and paralysis in infants and young children. J Appl Physiol.

[13] Dobbinson TL, Nisbet HI, Pelton DA, Levison H (1973). Functional residual capacity (FRC) and compliance in anaesthetized paralysed children. II. Clinical results. Can Anaesth Soc J.

[14] Serafini G, Cornara G, Cavalloro F, Mori A, Dore R, Marraro G (1999). Pulmonary atelectasis during paediatric anaesthesia: CT scan evaluation and effect of positive end-expiratory pressure (PEEP). Paediatr Anaesth.

[15] Trachsel D, Svendsen J, Erb TO (2016). von Ungern-Sternberg BS. Effects of anaesthesia on paediatric lung function. Br J Anaesth.

[16] Feldman JM (2015). Optimal ventilation of the anesthetized pediatric patient. Anesth Analg.

[17] Wanderer JP, Ehrenfeld JM, Epstein RH, Kor DJ, Bartz RR, Fernandez-Bustamante A (2015). Temporal trends and current practice patterns for intraoperative ventilation at U.S. academic medical centers: a retrospective study. BMC Anesthesiol.

[18] Kneyber MC (2015). Intraoperative mechanical ventilation for the pediatric patient. Best Pract Res Clin Anaesthesiol.

[19] Erranz B, Díaz F, Donoso A, Salomón T, Carvajal C, Torres MF (2015). Decreased lung compliance increases preload dynamic tests in a pediatric acute lung injury model. Rev Chil Pediatr.

[20] Díaz F, Erranz B, Donoso A, Carvajal C, Salomón T, Torres M, Cruces P (2013). Surfactant deactivation in a pediatric model induces hypovolemia and fluid shift to the extravascular lung compartment. Paediatr Anaesth.

[21] Feldman JM (2015). Optimal ventilation of the anesthetized pediatric patient. Anesth Analg.

[22] Peterson-Carmichael S, Seddon PC, Cheifetz IM, Frerichs I, Hall GL, Hammer J (2016). ATS/ERS working group on infant and young ChildrenPulmonary function testing. An official American Thoracic Society/EuropeanRespiratory society workshop report: evaluation of respiratory mechanics andFunction in the pediatric and neonatal intensive care units. Ann Am Thorac Soc.

[23] Kaditis AG, Motoyama EK, Zin W, Maekawa N, Nishio I, Imai T (2008). The effect of lung expansion and positive end-expiratory pressure on respiratory mechanics in anesthetized children. Anesth Analg.

[24] Cruces P, González-Dambrauskas S, Quilodrán J, Valenzuela J, Martínez J, Rivero N (2017). Respiratory mechanics in infants with severe bronchiolitis on controlled mechanical ventilation. BMC Pulm Med.

[25] Jonson B, Richard JC, Straus C, Mancebo J, Lemaire F, Brochard L (1999). Pressure-volume curves and compliance in acute lung injury: evidence of recruitment above the lower inflection point. Am J Respir Crit Care Med.

[26] Protti A, Maraffi T, Milesi M, Votta E, Santini A, Pugni P (2016). Role of strain rate in the pathogenesis of ventilator-induced lung edema. Crit Care Med.

[27] Amato MB, Meade MO, Slutsky AS, Brochard L, Costa EL, Schoenfeld DA (2015). Driving pressure and survival in the acute respiratory distress syndrome. N Engl J Med.

[28] Lucangelo U, Bernabé F, Blanch L (2005). Respiratory mechanics derived from signals in the ventilator circuit. Respir Care.

[29] Neto AS, Hemmes SN, Barbas CS, Beiderlinden M, Fernandez-Bustamante A, Futier E (2016). Association between driving pressure and development of postoperative pulmonary complications in patients undergoing mechanical ventilation for general anaesthesia: a meta-analysis of individual patient data. Lancet Respir Med.

[30] Wirth S, Artner L, Broß T, Lozano-Zahonero S, Spaeth J, Schumann S (2016). Intratidal recruitment/derecruitment persists at low and moderate positive end-expiratory pressure in paediatric patients. Respir Physiol Neurobiol.

[31] Rausch SMK, Haberthur D, Stampanoni M, Schittny JC, Wall WA (2011). Local strain distribution in real three-dimensional alveolar geometries. Ann Biomed Eng.

[32] Webb HH, Tierney DF (2003). Experimental pulmonary edema due to intermittent positive pressure ventilation with high inflation pressures. Protection by positive end-expiratory pressure. Am Rev Respir Dis.

