# Supplementary figures and images for: Positive end-expiratory pressure improves elastic working pressure in anesthetized children
Source: BMC Anesthesiol. 2018 Oct 24;18:151. doi: 10.1186/s12871-018-0611-8 (PMC6201576; doi:10.1186/s12871-018-0611-8)

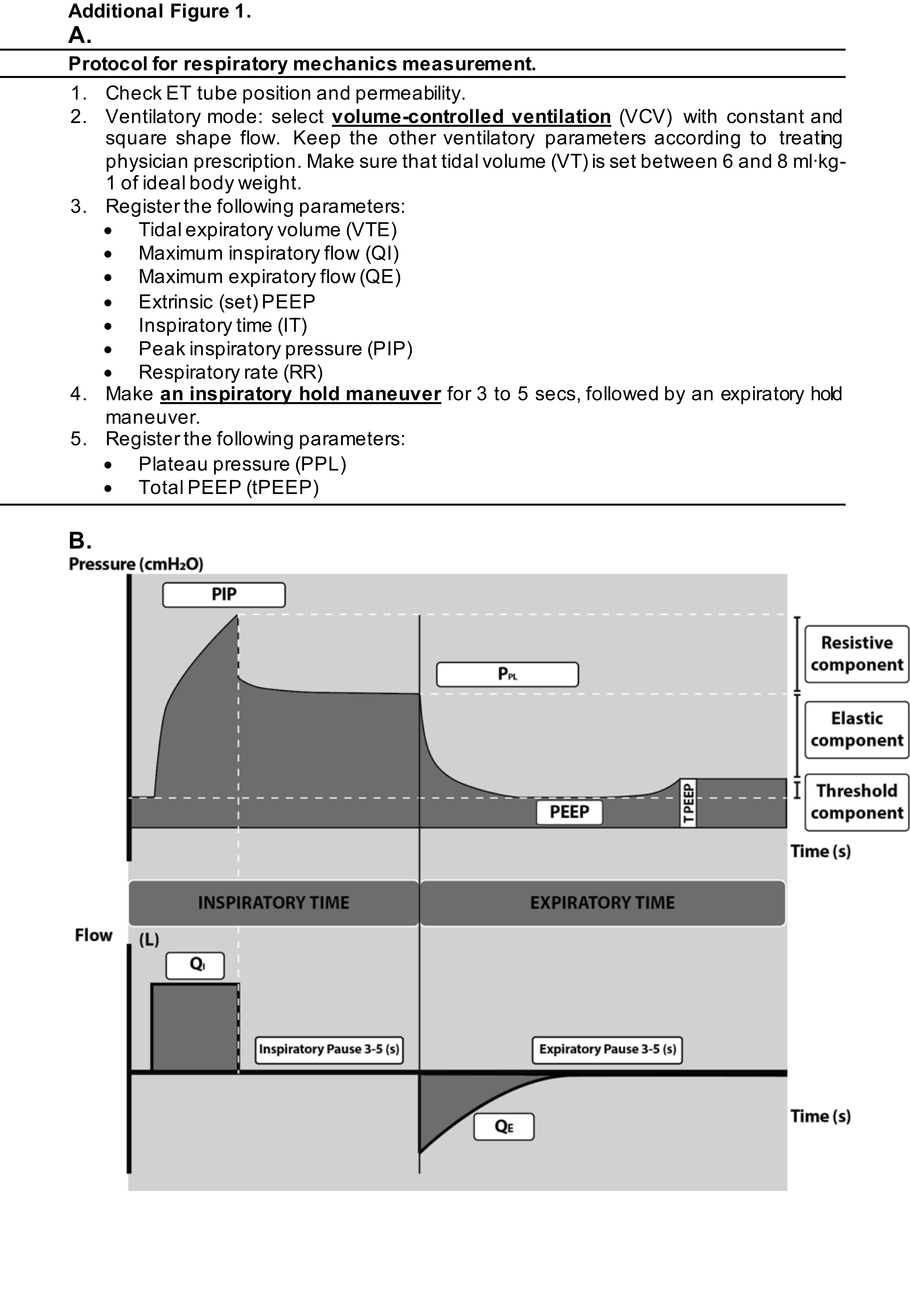

Supplement: Supplementary file 1 — Respiratory mechanics measurements. Panel A shows Respiratory mechanics measurement protocol. Panel B shows and illustration of Airway Pressure versus time and flow versus time curves during inspiratory and expiratory breathhold. The components of work of breathing, elastic and threshold are represented. (JPG 2451 kb) [file 12871_2018_611_MOESM1_ESM.jpg]
