# Supplementary material for: Positive end-expiratory pressure improves elastic working pressure in anesthetized children
Source: BMC Anesthesiol. 2018 Oct 24;18:151. doi: 10.1186/s12871-018-0611-8 (PMC6201576; doi:10.1186/s12871-018-0611-8)
Supplement: Supplementary file 2 — Formulas for estimation of lung mechanics in quasi - static conditions. (DOCX 15 kb) [file 12871_2018_611_MOESM2_ESM.docx]

**Additional file 2. Formulas for estimation of lung mechanics in quasi - static conditions.**

| C_RS_: Respiratory system compliance  ΔP: Driving pressure  iPEEP: Intrinsic PEEP  K_TI_: Inspiratory time constant  K_TE_: Expiratory time constant  Paw: Mean airway pressure  PEEP: Positive end-expiratory pressure (set)  PIP: Peak inspiratory pressure  P_PL_: Plateau pressure  Q_I_: Maximum inspiratory flow  Q_E_: Maximum expiratory flow  RawE: Resistance expiratory airway  RawI: Resistance inspiratory airway  tPEEP: Total PEEP  V_T_: Tidal volume | **Equation of Motion:**  Paw = V_T_ / C_RS_ + RawI · QI + iPEEP  **Resistive component**  RawI = (PIP - P_PL_) / Q_I_  RawE = (PPL - tPEEP) / Q_E_  **Elastic component:**  ΔP = P_PL_ - tPEEP  C_RS_ = V_T_ / ΔP  **Threshold Component:**  iPEEP = tPEEP - PEEP  **Time constants:**  K_TI_ = C_RS_ · RawI  K_TE_ = C_RS_ · RawE |
| --- | --- |
