# Supplementary material for: Positive end-expiratory pressure improves elastic working pressure in anesthetized children
Source: BMC Anesthesiol. 2018 Oct 24;18:151. doi: 10.1186/s12871-018-0611-8 (PMC6201576; doi:10.1186/s12871-018-0611-8)
Supplement: Supplementary file 3 — Correlations between changes in respiratory parameters and age and ideal body weight. (DOCX 15 kb) [file 12871_2018_611_MOESM3_ESM.docx]

**Additional file 3. Correlations between changes in respiratory parameters and age and ideal body weight.**

|  | **Age**  **(mo)** | **P value** | **IBW**  **(kg)** | **P value** |
| --- | --- | --- | --- | --- |
| **∆Q_E_** | -.401* | 0.028 | -.380* | 0.038 |
| **∆iPEEP** | 0.123 | 0.518 | 0.134 | 0.481 |
| **∆K_TE_** | .453* | 0.12 | .444* | 0.14 |
| **∆C_RS_** | 0.283 | 0.130 | 0.346 | 0.061 |
| **∆RawE** | 0.051 | 0.791 | -0.025 | 0.894 |
| **∆ΔP** | -0.172 | 0.364 | -0.216 | 0.252 |
| **∆ET** | 0.181 | 0.338 | 0.083 | 0.665 |
| **∆Q_I_** | 0.311 | 0.094 | 0.311 | 0.94 |
| **∆PIP** | 0.302 | 0.105 | 0.266 | 0.155 |
| **∆P_PL_** | 0.065 | 0.733 | 0.055 | 0.773 |
| **∆Paw** | 0.132 | 0.485 | 0.127 | 0.505 |
| **∆RawI** | 0.065 | 0.733 | 0.046 | 0.809 |
| **∆K_TI_** | 0.223 | 0.237 | 0.259 | 0.168 |
